# Two Cases of Fungus Hæmatodes

**Published:** 1841-10-23

**Authors:** A. W. Upshur


### MEDICAL EXAMINER.

DEVOTED TO MEDICINE, SURGERY, AND THE COLLATERAL SCIENCES.

No. 43.] PHILADELPHIA, SATURDAY, OCTOBER 23, 1841. [Vol. IV.

### ORIGINAL COMMUNICATIONS.

### *Two Cases of Fungus Hæmatodes.*

BY A. W. UPSHUR, M.D.

Gentlemen:—The unfrequent occurrence of Fungus Hæmatodes in this country has induced me to give you a very brief account of two cases which have recently come under my observation.

During the month of August, 1840, I was called, in consultation with my friend Dr. N., to see a lady who had been labouring under this painful disease for the space of nearly three years. The patient was a young and healthy individual up to the time of her attack. It first made its appeareance in the usual form of a small tumour, situated on the upper part of the thigh, near the groin. No attention was paid to it, however, and it gave no uneasiness, until some time during the summer of 1837, when, in consequence of an accident which she sustained at that that time, it began to increase in size, and to become slightly painful. She was, however, but little inconvenienced by it, and attended, as usual, to her domestic duties until a few months previous to her decease, when it attained to such an enormous size, and became so heavy and painful, as to keep her almost constantly confined to the bed.

I visited her for the first time in August, when the tumour had reached its worst form, having a very dark and gangrenous appearance, extremely vascular, and very irregular. It extended from the anterior superior part of the thigh to near the knee joint, and was about twenty-four inches in circumference. The patient at this time was extremely feeble and greatly emaciated. I was apprehensive that the circumstance of her being pregnant, and within a few weeks of her accouchment, would operate very much against her. Owing to the extreme vascularity of the fungus, I was inclined to think that the effort of delivery would produce a fatal hæmorrhage from the tumour. She was, however, safely delivered of a seven month's fœtus, without any apparent injury to the fungus. Prostration, however, shortly afterwards ensued, which terminated in death. We ordered nothing but cold applications to the part, knowing that the patient was far beyond the aid of human skill. A post mortem examination was proposed, but the heat of the weather produced such a rapid decomposition of the part, that it could not be effected.

*Case* 3.—I was called in haste, on the 21st of December last, in consultation with my friend Dr. H., to visit a negro belonging to Mr. C. The report of the messenger was, that "her knee had bursted, and that she was bleeding to death." Upon our arrival we discovered that the patient had, in reality, bled very copiously from an orifice (probably a ruptured vein) near the centre of the tumour, and but for a change of position, would probably have died from hæmorrhage.

The disease first made its appearance some time during the month of March, 1840, in the form of a small tumour near the knee joint, and was thought by Dr. H. to be a case of hydrarthrus, which disease it closely assimilated. It would not yield, however, to any treatment, and had, when I saw it, attained to nearly the size of a man's head, extending from near the middle of the thigh a considerable distance below the knee joint. The surface of the tumour on one side was considerably abraded, otherwise it was perfectly smooth, soft and spongy. A considerable quantity of fluid matter, resembling serum, was discharged from the abraded surface. The fungus, which was extremely painful at first, was greatly relieved by the hæmorrhage, and decreased considerably in size. In addition to the tumour on the knee, there were several others on the right side about the size of an egg, which were harder and less spongy to the touch. Had it not been for the existence of the tumours in the side, the great swelling of the limb, and the extreme debility of the patient, we should probably have had recourse to amputation, although with very little probability of success, even under the most favourable circumstances. Nothing, therefore, could be done, but to apply cloths wet in cold vinegar and water. In the course of a fortnight ulceration took place, which, in a few days, terminated in death.

The mode of treating this disease successfully remains yet to be discovered, and this I leave to those who are wiser and more experienced than myself.

